# Ultrafast Laser Excitation Improves LIBS Performance for the Analysis of Optically Trapped Single Nanoparticles Owing to Characteristic Interaction Mechanisms

**DOI:** 10.1021/acs.analchem.3c01376

**Published:** 2023-09-20

**Authors:** Clara Burgos-Palop, Pablo Purohit, Francisco J. Fortes, Javier Laserna

**Affiliations:** † UMALASERLAB, Departamento de Química Analítica, Universidad de Málaga, C/Jiménez Fraud 4, Málaga 29010, Spain; ‡ Niels Bohr Institute, University of Copenhagen, Blegdamsvej 17, Copenhagen 2100, Denmark

## Abstract

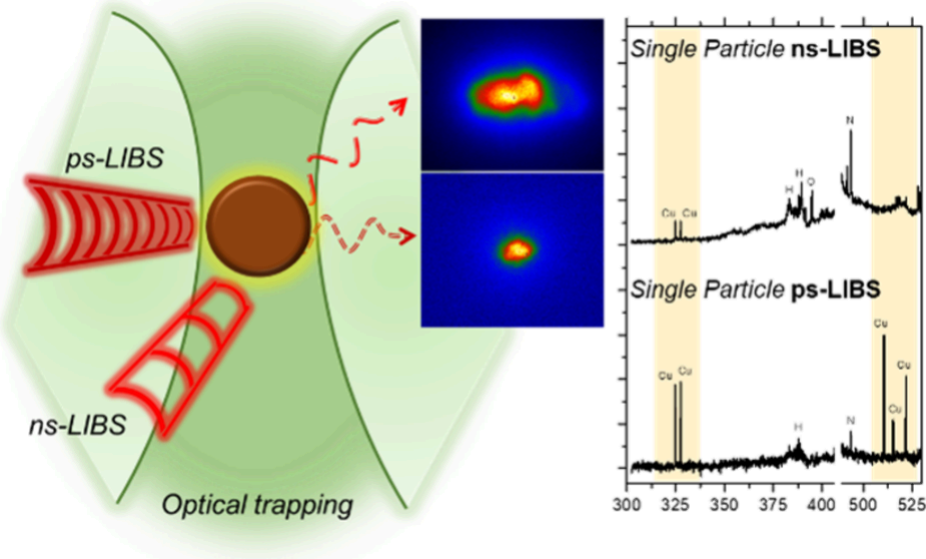

Owing to the exceedingly small mass involved, complete elemental characterization of single nanoparticles demands a highly precise control of signal background and noise sources. LIBS has demonstrated remarkable merits for this task, providing a unique tool for the multielemental analysis of particles on the attogram–picogram mass scale. Despite this outstanding sensitivity, the air plasma acting as a heat source for particle dissociation and excitation is a meddling agent, often limiting the acquisition of an accurate sample signature. Although thermal effects associated with ultrashort laser pulses are known to be reduced when compared to the widely used nanosecond pulse duration regime, attempts to improve nanoinspection performance using ultrafast excitation have remained largely unexplored. Herein, picosecond laser pulses are used as a plasma excitation source for the elemental characterization of single nanoparticles isolated within optical traps in air at atmospheric pressure. Results for picosecond excitation of copper particles lead to a mass detection limit of 27 attogram, equivalent to single particles 18 nm in diameter. Temporally and wavelength-resolved plasma imaging reveals unique traits in the mechanism of atomic excitation in the picosecond regime, leading to a deeper understanding of the interactions occurring in single nanoparticle spectroscopy.

The nanoinspection toolbox currently comprises a combination of new analytical solutions and classic in-bulk techniques adapted to target traits and processes at the nanometric scale specifically.^1−4^ Electrical and thermal conductivity,^5,6^ optical absorption, and localized surface plasmon resonance^7,8^ or surface reactivity^9^ are examples of magnitudes that can be measured using physicochemical methods reported in the recent literature. The finesse found in the design of nanomaterials and their applications implies the necessity of precisely knowing their behavior down to the single particle (SP) level in some cases.^4^ Laser-induced breakdown spectroscopy (LIBS) has been demonstrated as a powerful asset for the chemical characterization of single nanoparticles due to its capability to detect every constituent from an isolated multi-elemental sample in a single event.^10,11^ This fact implies the simultaneous identification of absolute quantities in the range from femtograms to attograms. Moreover, experiments are mostly performed in air at atmospheric pressure, thus simplifying the instrumental setup required.^12^ In SP-LIBS, pulse durations (τ) in the range of nanoseconds (ns-) have been the only option explored for particle dissociation and excitation of free atoms into emissive states.^10−12^ The underlying logic for this choice is the main mechanism reported in the literature for both processes: the plasma–particle interaction.^13,14^ This mechanism implies that, owing to mainly spatial reasons arising from either the location of the particle away from the laser focus or differences between the particle diameter and the spot size, the direct laser-particle interaction is of limited efficacy. Therefore, air molecules surrounding the isolated particle absorb most of the laser beam’s energy, which causes the onset of an air plasma. The air plasma then acts as a repository and cedes part of its energy to the particle in the form of heat, which is engulfed in the plasma. This rate-limited process coincides with mass transfer from the particle into the plasma. The free atoms are subsequently excited by the excess energy, and light emission follows. Thus, air plasma is the main source for particle dissociation and excitation. Plasma–particle interaction demands the high thermal effects associated with ns excitation to enhance the efficiency of both processes and improve the analytical performance of SP-LIBS. Recently, ultrafast excitation sources, with τ in the picosecond (ps*-*) or femtosecond (fs*-*) range, have gained relevance for a number of leading-edge LIBS applications.^15−17^ The advantages associated with ultrafast laser pulses include (i) a better spatially localized ablation and (ii) a significant reduction of thermal effects, both leading to reduced continuum and ionic emission of the sparked plasmas, thus resulting in improved detection of the signals associated with the excited species. Interestingly, despite these advantages, most applications still opt for nanosecond excitation since the reduction of thermal effects usually involves fewer spectral lines and cooler plasmas, resulting in an overall reduction of the emission intensity.^17−19^ LIBS inspection of single nanoparticles falls within the category of high sensitivity-demanding applications.^10−12^ In this context, exploring picosecond pulses for sample excitation as the intermediate case between the ns- and the fs- regime may unveil a powerful tool for enhancing the characterization of isolated nanoparticles. As the temporal window in which electron-heat conduction occurs is in the ps- scale,^20^ the use of laser pulses in this regime is likely to result in a reduction of the continuum emission and a less relevant presence of ionic species compared to ns- excitation, while still ensuring thermal effects to a certain extent. The potential benefits of ps*-* excitation could be further exploited in experimental setups designed explicitly for single-particle analysis such as electrodynamic balance^21−23^ or optical trapping.^10−12,24^ In both cases, liquid layers, if present at all, are evaporated by the action of either the electric field or laser-induced heating, hence allowing dry particles to interact directly with the plasma and boosting the efficiency of the process. As an example of the current relevance of LIBS-based SP techniques, OT-LIBS, a combination pioneered by our laboratory at the University of Malaga, has been then used to study micron-sized aerosols under a variety of scenarios.^25,26^

In the present work, we explore the use of picosecond laser pulses as the excitation source to perform the chemical characterization of single nanoparticles isolated in atmospheric pressure optical traps by LIBS. Results from NPs ranging from 25 to 70 nm in diameter are presented and compared to those yielded by identical particles under ns- excitation. Analytical figures of merit are discussed on the basis of the particular traits linked to each regime. The specifics of the ps*-* laser-induced plasmas-single nanoparticle interaction mechanisms are discussed with simultaneous temporally-resolved spectroscopy and imaging studies being performed for a deeper understanding of the phenomena taking place during particle atomization and their relation to the final recorded signal. Moreover, physical traits of the plasmas, such as the electronic temperature (*T*_e_), are calculated to thoroughly highlight the differences between excitation regimes and provide solid justifications for them. Results indicate improvements in the signal-to-noise ratios for the species herein inspected. The reduced interference of the air plasma enables the detection of neutral analyte lines not featured in nanosecond spectroscopy, suggesting picosecond pulses as a feasible means to characterize single nanoparticles in LIBS.

## Experimental Section

### Experimental Setup

**Figure 1:**
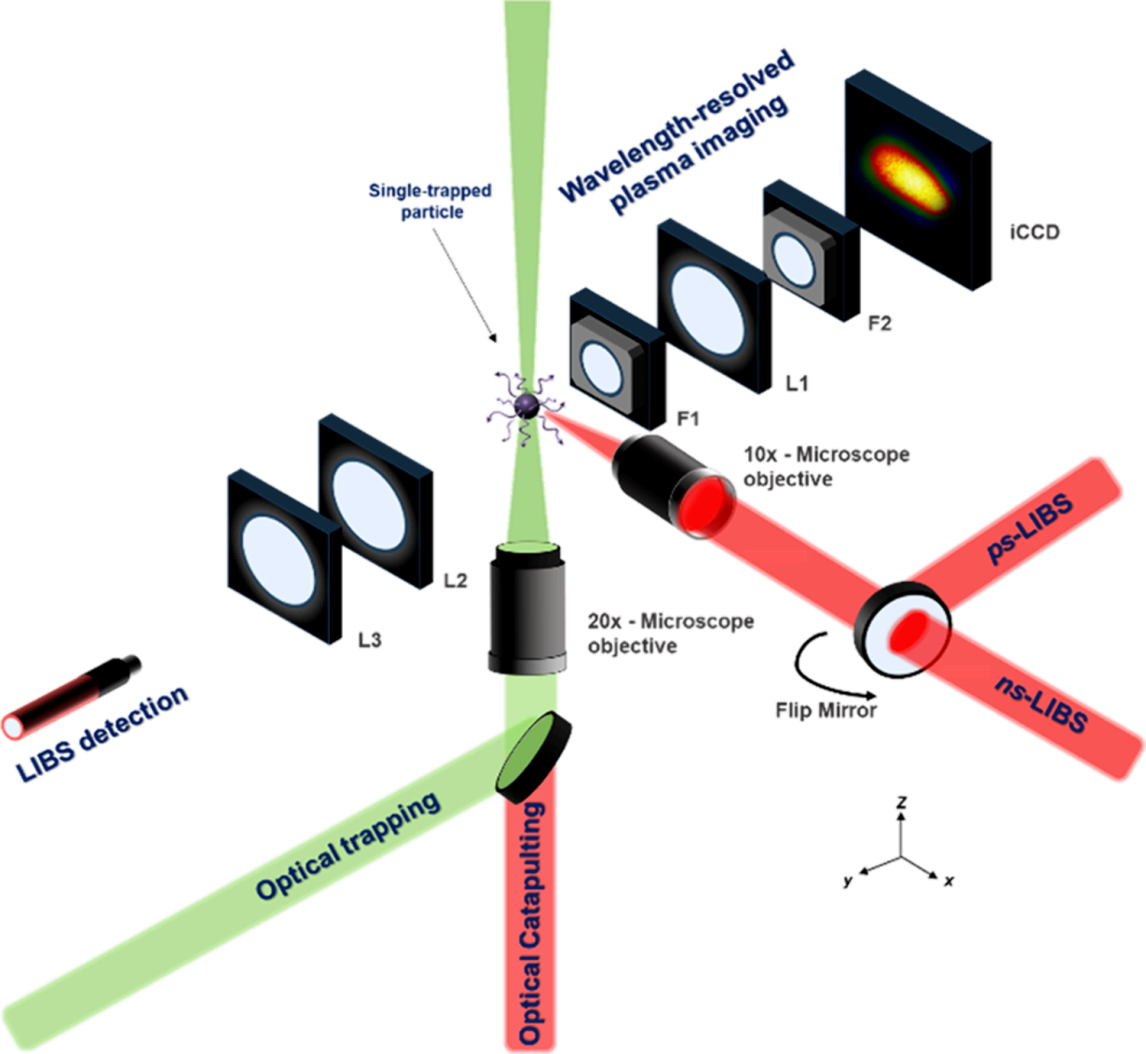
Experimental setup depicting the different excitation and detection lines comprising the λP-OC-OT-LIBS instrument. F1–2 are filters. L1–3 are lenses.

The in-depth description of the optical catapulting-optical trapping-LIBS (OC-OT-LIBS) experimental setup used in the present work can be found in previous studies.^10,11^ Details of the wavelength-resolved plasma imaging scheme are found in a previous study.^27^Figure 1 summarizes the setup and the full optical train for each excitation and collection lines. The figure also depicts the axes of the setup, therefore indicating the spatial location of each component. Briefly, this platform for the analysis of individual nanoentities integrates three lasers used for (i) the aerosolization of the material to be inspected, (ii) the optical trapping of individual micro- and nanoparticles, and (iii) sparking a plasma for the LIBS and plasma imaging analysis of the particle of interest. The workflow of the platform consisted of several steps. First, a Q-switch Nd:YAG laser (Spectron SL284; @1064 nm; 6 ns pulse width; 260 mJ/pulse) operating at its fundamental wavelength was focused through a long working distance microscope objective (Nikon; magnification 20×; 0.45 N.A.) toward the platform where the sample chamber was placed. In this way, the sample dry particles deposited inside the sampling chamber were catapulted into the aerosol form along the laser beam propagation direction without any mechanical contact. Coaxial to the catapulting laser and focused by the same objective, individual particles from the catapulted material were trapped by a CW laser (Lasertechnik; 300 mW output; 96 mW at the sample plane) operating at λ = 532 nm. The location of the optical trap and the position of the single-trapped particles were adjusted by manipulating the microscope objective along the *z*-axis. Light scattered by the isolated particle was recorded using an iCCD camera (Andor iStar DH334T, 2048 × 2048 pixels, Ireland) located in the *y*-axis for live-tracking of particles along the trapping axis. This camera was also used for regular and wavelength-resolved plasma imaging. For LIBS analysis, the entire system was aligned to a (0, 0, 0) coordinate dictated by the intersection of the analyzing laser focus and the trapped particle. To evidence this position, an image from a particle-less air plasma was acquired using the iCCD camera, with the (0, 0, 0) spot being the center of the plasma core. Particle excitation was performed using either a ps*-* or a ns*-* laser source. The ultrafast laser, with a pulse width in the picosecond range (Q-switch Nd:YAG; @1064 nm; 100 ps pulse width; 100 mJ/pulse), was guided by two high reflectivity mirrors and focused toward a 10× microscope objective (Thorlabs; 15 mm working distance; 0.25 N.A.) to form a plasma on the single trapped particle. Nanosecond excitation was performed by using a Q-switched Nd:YAG laser working at its fundamental wavelength (1064 nm) and a pulse width of 6 ns. This pulse was directed into the same focusing objective with a set of two mirrors. For LIBS detection, the plasma light was collected by a pair of plano-convex lenses (UV-FS; 2-inch diameter; 100 mm focal length) to focus the light on the tip of an optical fiber (Avantes; 2 m long; 0.22 N.A.; 600 μm diameter), connected to a time-integrated Czerny-Turner spectrometer (Avantes). A 1200 lines/mm holographic diffraction grating was employed to record lightemission in the 300–550 nm range. The same collection line was used for the time-resolved LIBS analysis. In this operation mode, the optical fiber was connected to a time-resolved Czeny-Turner spectrometer (Shamrock SR-303i; 250 mm focal-length; grating of 300 lines mm^–1^) using an iCCD camera as detector (Andor iStar DH734, 2048 × 2048 pixels, Ireland). Specific temporal conditions for time-resolved analysis are presented in the appropriate subsection. Synchronization between lasers was externally controlled by a pair of pulse and delay generators, which allowed control of the interpulse delay and data-acquisition parameters.

For simultaneous wavelength-resolved plasma imaging, the light was focused on an iCCD camera. First, the plasma light (in the *xz* plane) passed through a short-pass filter (F1) that reflected light outside the 310–375 nm window. Then, the light was focused by a biconvex lens (UV-FS; 1 inch diameter; 50 mm focal length) into a second wavelength-discrimination filter (F2) to exclusively allow light at 324.75 nm to reach the detector.

### Samples

Qualitative portions (typically, a few micrograms) of standard copper nanospheres (MkNano, Canada) of 25 (±3.7), 50 (±7.5), and 70 (±10.5) nm in diameter were used for the experiments. To prepare the sampling chamber, sample powder was deposited directly onto a 200 μm thick glass slide and covered with 10 mm path length quartz cuvettes. The amount of deposited sample was small enough to generate low-density solid aerosols that facilitate the inspection of single nanoparticles. Empty sampling chambers were used for recording blank air plasmas. To prevent diffusion of the sample into the air, the cuvette was glued to a glass support. The protocol for sample handling was carefully controlled to obtain a homogeneous and reproducible particle distribution inside the cuvette. The cuvette was utterly free from contamination, and its manipulation was performed using gloves. Sample inspection was performed at room temperature in air at atmospheric pressure.

### Data Processing

Acquired data were checked to verify that they sourced from a single particle before undergoing further processing steps. A sorting scheme, based on the linear relation between LIBS signal and inspected mass, was applied to the sets following the process described in ref (10). Briefly, the first step was to discern the events present in raw LIBS data sets tied to particle clusters. Such events were easily identified upon plotting the intensity of the 324.75 nm Cu(I) line owing to their signal being up to an order of magnitude larger than that along the trend patterned by the rest of the data. Moreover, if any, LIBS spectra with a signal-to-noise ratio (SNR) value below 3 were discarded for data analysis due to them belonging to particles located to the periphery of the optical trap. The net intensity of the remaining events was averaged as μ_events_. To establish the upper limit from which the event cannot be linked to a single NP, we used μ_events_ and its standard deviation (*s*_events_): μ_events_ + 3*s*_events_. The lower I limit, to establish the minimum signal that could be attributed to a single NP at the center of the optical trap, was set as 3*s*_background_ (where *s*_background_ is the standard deviation of the background in the 315–320 nm spectral range). This decision was inspired by the common spectroscopic criteria of signals requiring SNR > 3 to be considered as detected.

With regard to plasma imaging, one of two different modes was used: time-resolved imaging and λP-imaging. The latter mode, reported in ref (27), combined time with wavelength resolution, hence resulting in images containing solely light coming from the particles. In our case, we targeted the emission line of Cu(I) at 324.75 nm for λP imaging, given its high intensity. To obtain time-resolved images, used for diagnosis, the filters shown in Figure 1 were removed to allow photons of all wavelengths to reach the iCDD camera. For these images, the delay time (*d*) between plasma onset and image recording was adjusted as needed. The integration time (*w*) was modified as well according to the delay value, although being generally kept between 5 and 100 ns^24^ with a MCP gain set to 0. *F*1 and *F*2 were placed along the *y* optical axis of the setup when λP images were acquired. In this case, the temporal setting for acquisition was fixed to 100 ns, delay time was incremented in 100 ns steps, and the gain was set to 0 again. To facilitate monitoring the evolution of the particle signal in λP, the images comprised within a series were normalized to the intensity maxima of the set, hence ensuring an identical scale for every image.

## Results and Discussion

### Qualitative Comparison of Spectra and Plasma Images Generated with Nanosecond and Picosecond Pulsed Lasers

**Figure 2:**
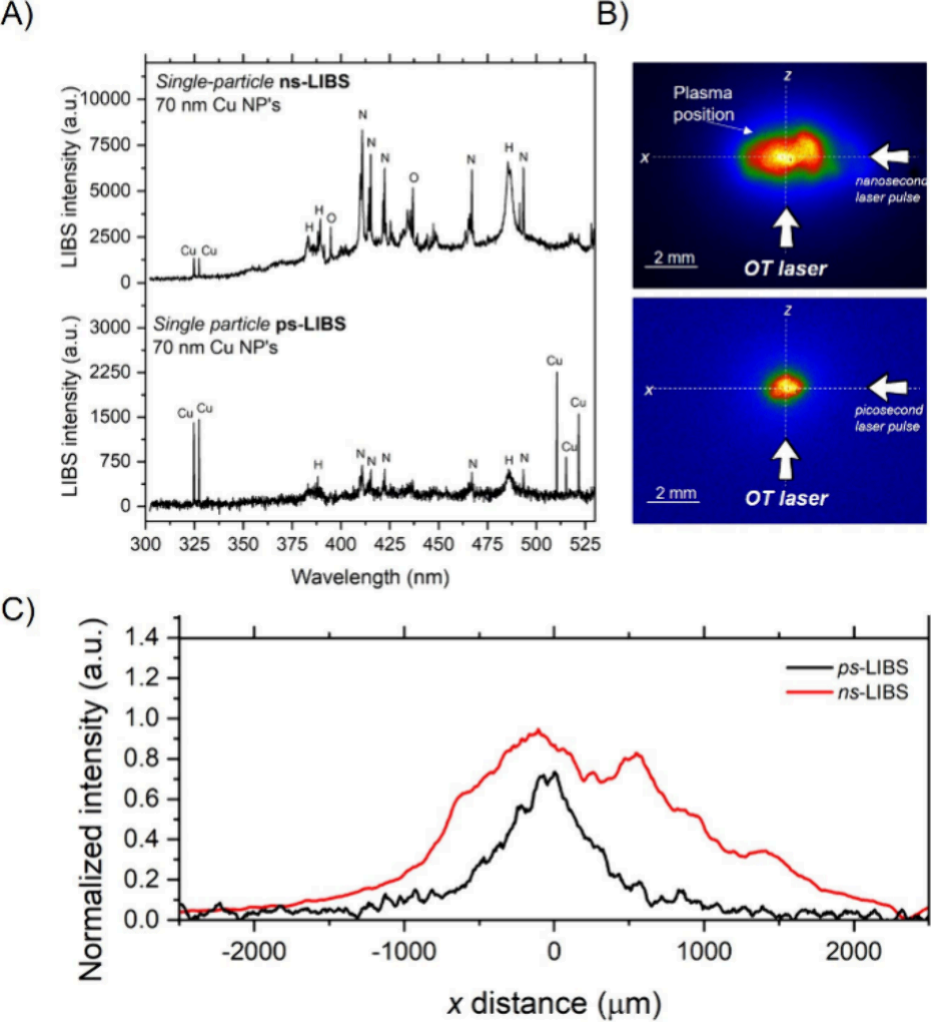
(A) Example spectra yielded by single 70 nm Cu NPs upon excitation with a ns*-* (top) or a ps*-* (bottom) laser pulse. (B) Typical morphology and dimensions for plasmas of 70 nm Cu NPs produced by fast (left) or ultrafast (right) pulses. (C) Length of the plasma plumes shown in Figure 2B, quantifying the size collapse observed in the images.

As mentioned in the Introduction section, due to the different excitation mechanisms sparking the plasma in picosecond and nanosecond LIBS, ultrafast laser pulses were expected to yield major spectral changes as well as to result in other relevant plasma traits such as size or shape. To highlight key changes arising from the temporal length of the laser pulse employed, we compared LIBS spectra and plasma images produced under each temporal regime for single Cu particles of 70 nm diameter. Figure 2A shows example LIBS spectra recorded at an identical time after plasma onset. This time was set to *d* = 5.28 μs following previously reported optimum values for the inspected samples.^10^ From these results, the most substantial change among spectra corresponds to the presence of the emission lines of Cu(I) at 510.50, 515.29, and 521.74 nm, which exclusively appear in ps-LIBS. Along the 510–521 nm triad, the emission lines at 324.75 and 327.40 nm, which are the only Cu lines featured in nanosecond excitation, were also observed. The appearance of these emission lines hinted at a potential advantage of using ultrafast excitation for SP elemental characterization: enhanced qualitative capabilities owing to a greater number of analyte-related spectral signatures. Two intertwined arguments may explain the presence of these lines, previously only seen in bulk and aerosol Cu-containing sample analysis.^28^ On the one hand, ps spectra featured a significant reduction of the intensity value of the spectral background. For the same energy density value, *F* = 520 J/cm^2^ (fluence, *F* ≡ J/cm^2^), the background emission was ca. 1500 a.u. in ns whereas it was close to the baseline level in ps excitation. The result agreed with the initial hypothesis of reduced influence of continuum and ionized air in ps*-* owing to less thermic effects and more spatially confined excitation.^29^ An additional qualitative fact extracted from spectra in Figure 2A is the difference in the full width at half-maximum (FWHM) for ns- and ps-LIBS.

The plasma images corresponding to the spectra mentioned above are displayed in Figure 2B. In the pictures, the size of the laser-induced plasma is observed to considerably decrease for ps-LIBS. This observation can be attributed to the characteristics of the laser pulse used for its formation. The direct interaction of the plasma with the incident nanosecond laser pulse leaded to further heat exchange with the medium and caused the plasma to expand instead of being confined around the laser focus. Conversely, due to the short τ, the interaction between the plasma and the tail of the laser pulse is much smaller in ps-LIBS. In this case, laser-induced heating of the air plasma occurred to a much lower extent. The lack of laser beam absorption implied that no excess energy could be transferred into the concomitant air molecules for expansion, and the ps*-*plasma was confined to a smaller spatial region. Enhanced air molecule dissociation rate and excitation is further evidenced by the larger intensity of the atomic lines associated with air in ns-SP-LIBS. The spatial constraint of the ps*-*plasma can also promote the aforementioned second effect, which leaded to the emergence of the 510–515–521 Cu lines: more efficient excitation of free Cu atoms. In SP-LIBS, owing to the plasma–particle interaction being the predominant mechanism for atom promotion into emissive state, low-lying excited levels (*E_k_*) are known to be populated preferentially as they are easily accessible from an energy point of view.^11^ Once these levels are saturated, other transitions populate as long as the plasma can give enough energy to the free atoms. In the case of Cu, the lines at 324.75 and 327.40 nm involved the ground level (*E_i_* = 0 eV) and excited states of lower energy than those participating in the 510–521 nm triad. The energy values for the copper transitions are listed in Table 1. As seen, the *E_i_* required to be populated for the transits at 515.29 and 521.74 nm is the *E_k_* levels of the 324.74 and 327.40 nm lines. In fact, *E_i_* (515.29) = *E_k_* (327.40) and *E_i_* (521.74) = *E_k_* (324.75) = *E_k_* (510.50). The inter-relationship between the levels suggested that the lines corresponding to the visible zone require a higher energy input for the Cu atoms to reach the *E_k_*s. In ps-excitation, the reduced plasma volume translated into the energy from the laser pulse being highly concentrated and reduced contact with air molecules. Moreover, the intensity profiles in Figure 2C also prove the smaller energy intake by air in the case of ps-excitation. Using both profiles, we calculated a reduction to 40.7% in size of the plasma plume, with its length being reduced from 2.7 mm in ns to 1.1 mm in ps. The area of the picosecond plasma was reduced by 24.3% when compared to the ns-plasma. Since energy is transferred in the form of heat, it is reasonable to conclude that the excess energy remaining after particle dissociation was invested in the Cu atoms engulfed in the air plasma, given their close contact with this energy reservoir. A fact supporting this claim is that, as in the example provided in Figure 2A, the 324–327 nm pair of lines was often saturated, as evidenced by the intensity ratio aspect observed between lines, which was different from the ca. 2:1 ratio expected from bibliographical data.^28^ Therefore, the closer localization to the particle in conjunction with a reduced spectral background favored the recording of the 510–521 nm triad in the spectrum. Overall, these results suggest that picosecond pulses are a powerful tool for eliminating spectral interferences and overlapping during single nanoparticle analysis.

**Table 1:** Energy Values for the Levels Involved in the Cu Lines Featured in Both ns- and ps- Spectra

line (nm)	energy of the lower level *E_i_* (eV)	energy of the upper level *E_k_* (eV)	energy gap (Δ*E*) (eV)
324.75	0	3.82	3.82
327.40	0	3.78	3.78
510.50	1.39	3.82	2.43
515.29	3.78	6.20	2.42
521.74	3.78	6.20	2.42

### Ultrafast Laser Pulses for the Quantitative Inspection of Single Nanoparticles

LIBS emission directly depends on the particle mass incorporated into the laser-induced plasma and the efficiency of this entity in exciting free atoms. To evaluate the sensitivity, we assessed their influence in the LIBS study of single Cu nanospheres 25, 50, and 70 nm in diameter. For this, laser events were acquired for each particle size, and the intensity of the Cu(I) emission line at 324.75 nm was tracked. The data processing protocol presented in the Experimental Section was implemented to ensure that each laser event exclusively corresponded to a single trapped particle instead of agglomerates.

**Figure 3:**
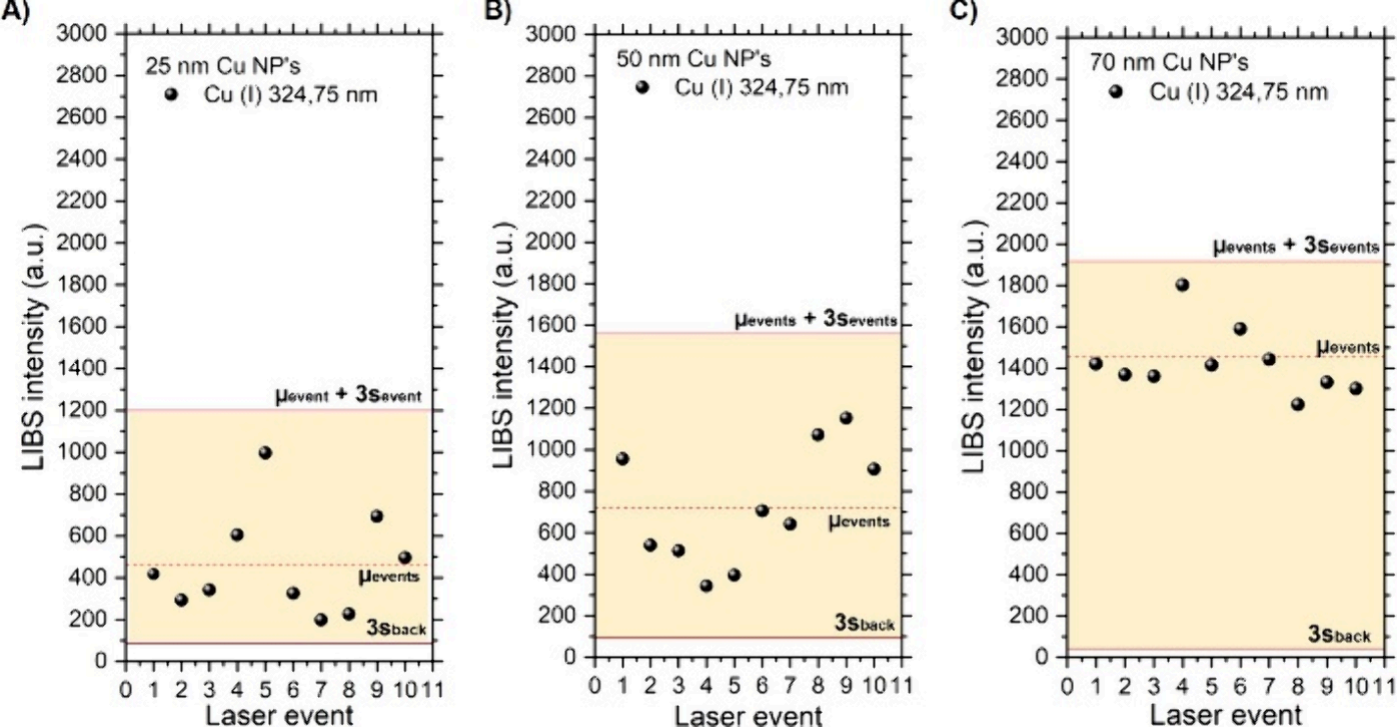
Sorting method applied to example picosecond data sets for each of the particle diameters inspected herein: (A) 25 nm, (B) 50 nm, and (C) 70 nm. The average intensity calculated herein was then used for further calculations.

**Figure 4:**
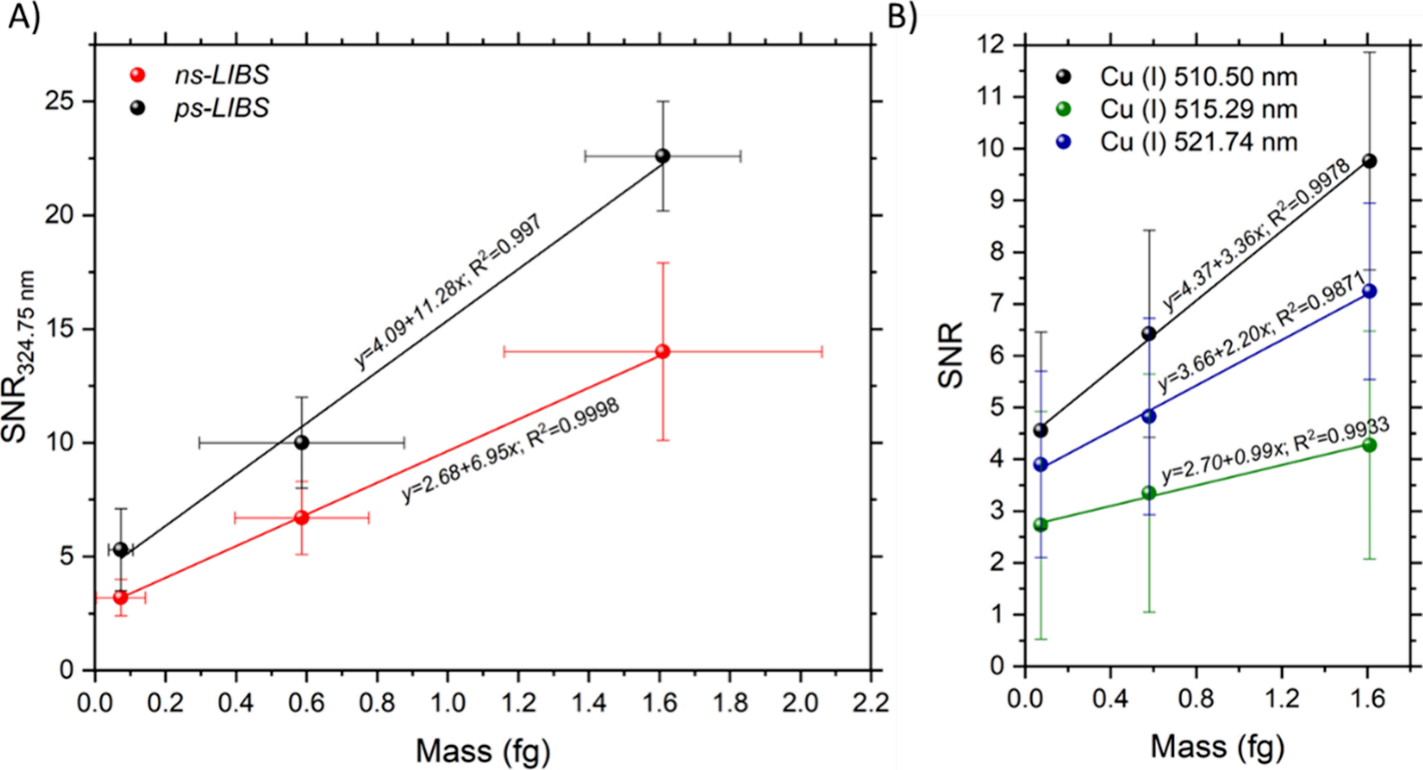
(A) Average SNR for 25, 50, and 70 nm in diameter after applying the sorting scheme described in the data processing section to sets acquired using ns*-* and ps*-* excitation plotted against each particle respective mass. The linear fits provide evidence that the use of the SNR values can enhance the performance of ps*-*LIBS for quantitative applications by taking advantage of the reduced noise in this excitation regime. From the *x*-error bars, we found that experimentally calculated mass dispersion for each particle size matched that specified by the manufacturer. This reinforced the exactitude of the OC-LIBS approach in the working diameter range. Moreover, the bars verified that threshold values calculated in Figure 3 were well-suited for the inspected samples and the proportionality between the readout and particle mass. (B) Average SNR for the three Cu(I) Vis lines extracted from the same spectra used to plot Figure 4A. These spectral signatures, which were only detectable using picosecond excitation, also showed a linear correlation with respect to the mass of the NPs. It is worth highlighting that only the line at 510.50 nm showed consistent SNR above 3 for every spectrum considered, hence making it the a priori best option for NP inspection. Error bars in the *x* axis for data in Figure 4B are provided in Figure S1 in the Supporting Information

Figure 3 illustrates the sorting method for data sets of different particle sizes, according to the above-mentioned criteria. As shown, for the case of study, none of the events fell outside the limits indicated so that they could be attributed to SPs and used for further evaluation. The averaged intensity, μ_events_, when using a picosecond laser pulse as dissociation and excitation source was μ_events_ (70 nm) = 1425 a.u.; μ_events_ (50 nm) = 822 a.u.; and μ_events_ (25 nm) = 459 a.u., respectively. Regarding signal variability, we observed a substantial increase for smaller particle sizes. The relative standard deviation (RSD) was 11% for the 70 nm NPs, 34% for the 50 nm NPs, and 53% for the 25 nm NPs. Relevant quantities, resulting from the classification of data produced under both regimes, are summarized in Table 2. It must be noted that, according to the manufacturer’s data, samples presented an inherent size dispersion of approximately 15%. Hence, given the cubic proportion between the particle size and mass (*m* ∝ *r*^3^), a slight variation in size at these levels can translate into significant mass difference from one event to another. In the case of the 25 nm particles, the mass spread reaches 43%, a value consistent with the reported signal variability. To evaluate how the ps-LIBS signal correlated to the particle size, the SNR for each particle diameter (obtained from the sorting presented in Figure 3) was plotted versus particle mass. The choice of utilizing the SNR to calculate the limit of detection for single Cu NPs in picosecond excitation was motivated by the background reduction observed for this regime. This approach has been used before for laser-based methods maximizing signal-to-noise ratio results due to the direct connection of LOD and SNR.^30^ Results are plotted in Figure 4A. The average mass for the three NP sizes featured in our experiments was 1.61, 0.59, and 0.07 fg for the 70, 50, and 25 nm diameters, respectively. For comparative purposes, data from ns-LIBS studies are also plotted in the graph. A total of 10 laser events from each excitation sources were considered for data processing. As seen, Figure 4 confirms that the linear proportionality between intensity and mass characterizing single-particle ns-LIBS^10,11^ is also found when using picosecond pulses

**Table 2:** Analytical figures of merit for *ns*- and *ps*- excitation in particles of different size.

	*ns*-LIBS			*ps*-LIBS		
	25 nm	50 nm	70 nm	25 nm	50 nm	70 nm
μ_events_^a^ (a.u.)	508	1011	2125	459	822	1425
RSD^b^ (%)	25.0	25.0	30.1	53.0	34.0	11.0
3s_background_^c^	160	150	151	86	82	63
SNR^d^_324.75nm_	3.3	6.7	13.8	5.3	10.2	22.1

a Averaged intensity at Cu (I) 324.75 nm for the inspected laser events.

b Precision expressed as RSD (%) of the spectral line intensity.

c Standard deviation of the background signal either side of the spectral line.

d Signal-to-noise ratio calculated from equation:

The respective linear fit equations and *R*-squared values were ps-LIBS (*y* = 11.15*x* + 3.89; *R*^2^=0.998) and ns-LIBS (*y* = 6.58*x* + 2.89; *R*^2^ = 0.9998). As commented above, picosecond excitation was found to lead to a significant reduction of the spectral background and, consequently, to improved signal-to-noise ratios. Table 2 also summarizes the SNR values calculated for the ps*-* and the ns*-* data sets. Improved ratio values for ps-LIBS translated into a steeper slope, hence implying that SNR values can be used to enhance the sensitivity of this regime for quantification. In the case of the ultrafast regime, this observation is tied to the reduced influence of continuum and ionized air caused by the reduced thermal effects and more spatially confined excitation, which imply a general background collapse. Quantitatively, this translated into a better performance of ps*-*pulses for originating more evident analyte-related signatures in LIBS spectra. As reported in previous studies,^10,29^ the observed linearity suggests complete atomization of the particles in the laser-induced plasma since no asymptotic value (characteristic of incomplete dissociations) was reached.^31^ The limits of detection in ps*-* were estimated by the equation, , where *C* is the mass of the nanoparticle, RSD_b_ is the relative standard deviation of the blank, and SNR is the signal-to-noise ratio. To perform this calculation, the data corresponding to the NPs of 25 nm were chosen since they were the lower inspected mass. From here, the calculated LOD for single copper nanoparticles using picosecond pulses as the excitation source was 27 attograms. To fully exploit the advantages of using ps excitation, we explored the use of the ps-exclusive Cu(I) Vis lines, with data extracted from the same spectra used in Figure 4A, for calculating the LOD. Results are plotted in Figure 4B. We found that identical to the 324.75 nm signal, the three lines featured a linear correlation with particle mass, albeit with lower SNRs and reduced slopes. Interestingly, the line at 510.50 nm, which features the lowest relative intensity out of the three according to literature, featured the steepest slope. Furthermore, this was the only line to systematically comply with the detection criteria of featuring SNRs above 3. Again, connecting to how the energy of the levels involved in the transit affects the collected spectra, we link this result to the easier accessibility of the *E_i_* and *E_k_* of the 510.50 nm line. Based on the 510.50 nm line, we calculated the LOD for Cu in the Vis range to be 29 attograms. Therefore, ps-SP-LIBS does not only featured more spectral signatures than conventional ns-SP-LIBS but also allowed the use of those lines for quantification without compromising the detection power of the approach. This can be especially useful in the event of spectral interferences or if the a priori preferred signature is prone to self-reversal, as can be the case for the 324.75 nm Cu(I) line under some interrogation scenarios.^28^ The LOD obtained indicated that the detection power of the OC-OT-LIBS technique was also in the range of attograms when using ultrafast laser pulses.

### Evolution of the Chemical Composition and the Physical Traits of SP Picosecond Laser-Produced Plasmas in Time

**Figure 5:**
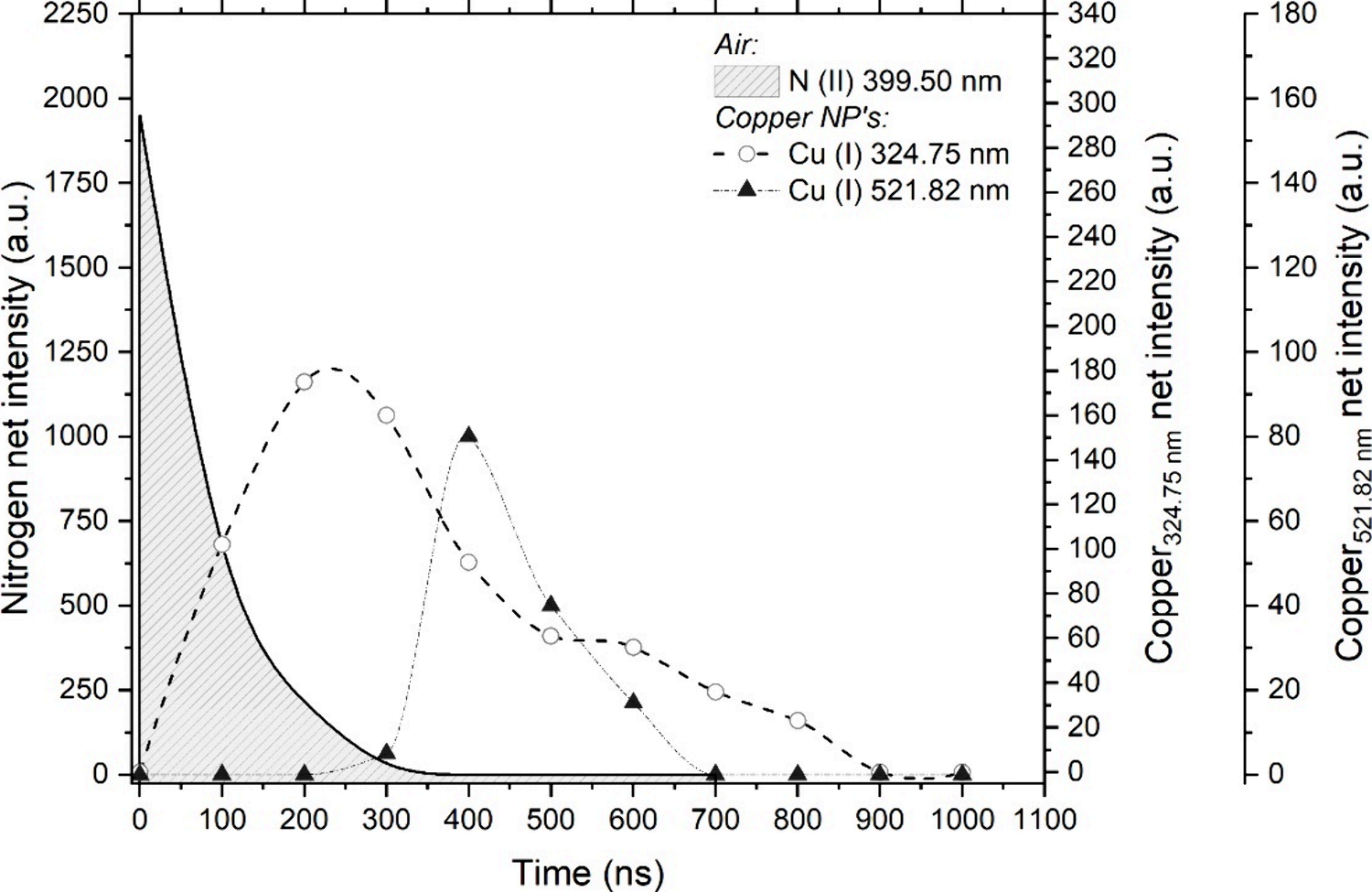
Temporal evolution of the N(II) line at 399.50 nm and Cu(I) lines at 324.75 and 521.82 nm using ps*-* laser pulses. It is important to point out that the Vis line only appeared after the emission at 324.75 nm peaked and started declining. This implies that the involved transit was populated as the *E_k_* level for the UV became empty.

**Figure 6:**
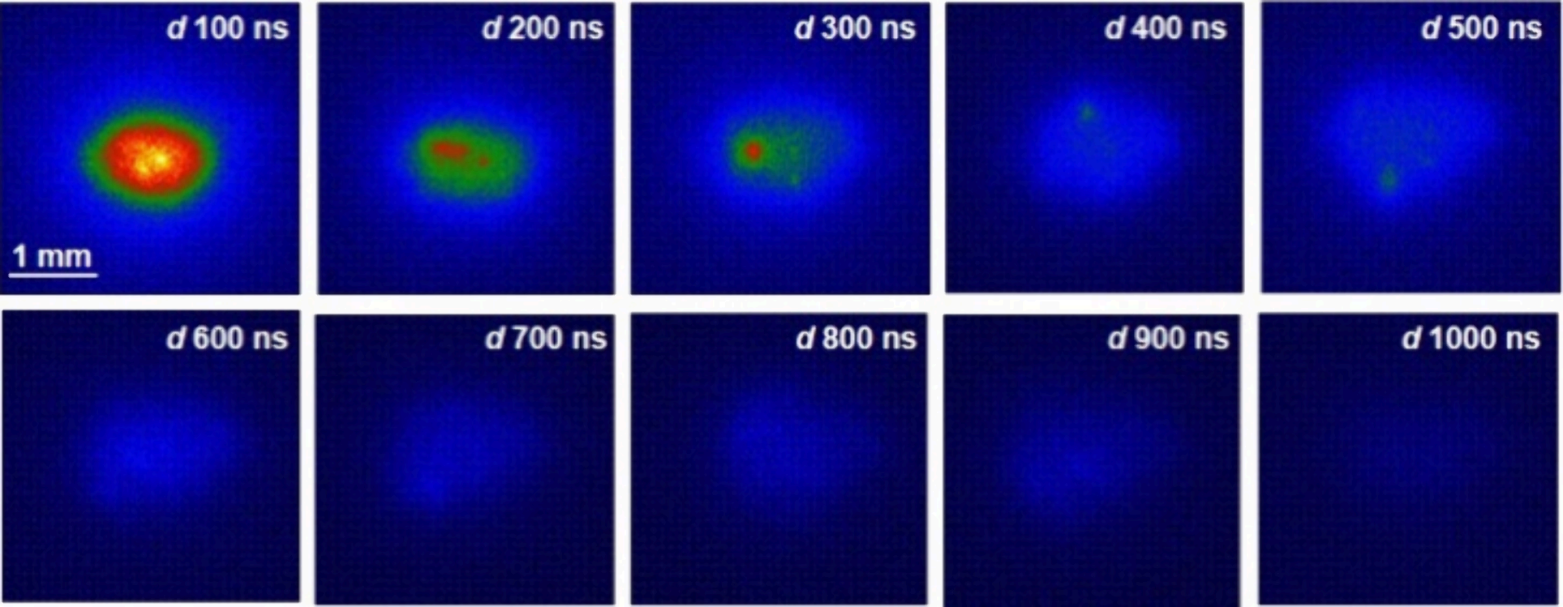
Temporally and wavelength-resolved plasma imaging of individual 70 nm Cu nanoparticles. At early delays, the core of the plasma is populated by electrons, producing unspecific emission of light. As the Bremsstrahlung radiation was extinguished, light from the line at 324.75 nm became observable. The particle contribution was randomly displaced from the center of the optical trap as a consequence of momentum transfer as it dissociated. Particle signals merged with the background at about 600 ns, in agreement with results recorded by LIBS.

To further investigate the relationship between the lifetime of the plasma produced using ps*-* pulses and the plasma–particle interaction, the evolution of the plasma chemical composition and the plasma temperature (*T*_e_) was studied. To do so, the spectral signal of Cu(I) 324.75, and Cu(I) 521.82 nm in 70 nm NPs was monitored by time-resolved spectroscopic and imaging analysis. Figure 5 presents the LIBS net intensity of the above-mentioned lines in the range 0–1000 ns. The integration time was kept at 100 ns. Each point in the graph is the average of three single-particle laser events. As previously discussed, the air plasma plays an important role as the energy source for particle dissociation and excitation. As shown in Figure 2, the air plasma resulted in prominent features in the recorded ns-LIBS spectra, whereas its presence was significantly reduced in ps*-* results. For this reason, the emission line of N(II) at 399.50 nm was monitored. As seen in Figure 5, the air signal (represented by nitrogen) rapidly decreased and extinguished at approximately 350 ns. Therefore, most of the interaction between both entities took place in the early plasma stages. From 0 to 100 ns, the particle absorbed energy from the laser-induced plasma to dissociate and promote the cleaved atoms to excited levels. Thus, the drop in the nitrogen signal translated into the appearance of the copper line at 324.75 nm at 100 ns. As expected from such an exiguous analyte source, the signal was transient-like, soon reaching its maximum value at 250 ns. In agreement with statements on preferential population of low-lying levels, only once the excited state of the 324.75 nm line reached its maximum intensity (as evidenced by the signal intensity recorded between 200 and 300 ns), the transit at 521.82 nm began to arise. This observation also implied that, coherently with the rate-limited energy transfer excitation mechanism proposed for plasma–NP interaction,^13^ the plasma was in a nonequilibrium situation until maximum population of the Vis lines as Cu atoms’ temperature were in constant increase. On a related note, the successive appearance of the Cu signals, with the lines requiring less total excitation energy emerging first, was also a consequence of the interaction mechanism. In this case, the processes of particle atomization and free atom excitation compete during early plasma lifetime, causing the UV line to be detectable at earlier time. As the particle disintegrates, access to the energy stored in the laser-produced plasma is facilitated for the free atoms, hence enabling the transitions between the levels involved in the 521.82 nm line. This discussion is supported again by the experimental time lapse found for the detection of both lines shown in Figure 5. Furthermore, the absence of energetic input from the laser pulse is the main explanation for the short plasma lifetime with signals vanishing around 900–1000 ns. In contrast, persistence of ns- plasmas can reach up to milliseconds.^19^ A major conclusion to be extracted from this study is that the picosecond plasma contained mostly copper atoms instead of air, as in the case of ns-LIBS, which is consistent with the smaller plasma volume observed in Figure 2B. Plasma chemistry evolution reinforced the ideality of ps*-* excitation for qualitative identification of the inspected particles. Moreover, the emission profile indicated the need for longer integration times to fully exploit the signal yielded by NPs by using ps- pulses. However, gate widths should be consistent with the short period over which particles produce photons and remain in an approximate interval of 100 ns < *d* < 1000 ns. Wavelength-resolved plasma imaging was conducted alongside LIBS studies to further understand, describe, and validate the phenomena occurring during plasma–particle interaction. As described in the Experimental Section, λP imaging collects photons at 324.75 nm simultaneously to LIBS analysis. For comparative purposes, images and LIBS spectra were acquired under the same temporal conditions. The integration time was also kept at 100 ns for λP imaging. Figure 6 shows the acquired chemical images of copper at 324.75 nm in the time frame of 0–1000 ns. As observed, at 0 ns, a bright circular core was formed at the particle position, i.e., at the beam focus of the ps*-* laser. This core exhibited signs of fragmentation and diffusion, as there was an emission gap at its center and high intensity pixels scattered around the center. The explosion of the particle as a consequence of exposure to high energy in early plasma lifetimes explains the traits observed in the image. It is also worth noting that unspecific radiation, which the optical filters could not discriminate, seemed to contribute to the regions of lower intensity in the image. As a result of the momentum induced from the explosion and diffusion of atoms while relocating to colder regions of the plasma, particle displacements were observed at around 100–200 ns. Although the nanoparticle remains closer to the plasma core in ps-LIBS, the behavior is similar to that previously reported using nanosecond excitation.^27^ At 300–400 ns, the particle was found to have reached the plasma periphery. The emission was limited to a decaying core, with no photons coming from the diffusion atomic cloud. From this point, the Cu signal was diluted in the spectral background until full extinction. These results are consistent with the temporal evolution of the Cu(I) line at 324.75 nm observed in Figure 5. Wavelength-resolved imaging analysis showed that, under ps*-* excitation, individual NPs also undergo a process of heat absorption from the plasma, followed by mass transfer to the plasma as the particle exploded due to overheating.^32^ An important difference found in the ultrafast regime is the earlier spawning of the particle signal and its faster decay, consequently, reducing the plasma lifetime.

**Figure 7:**
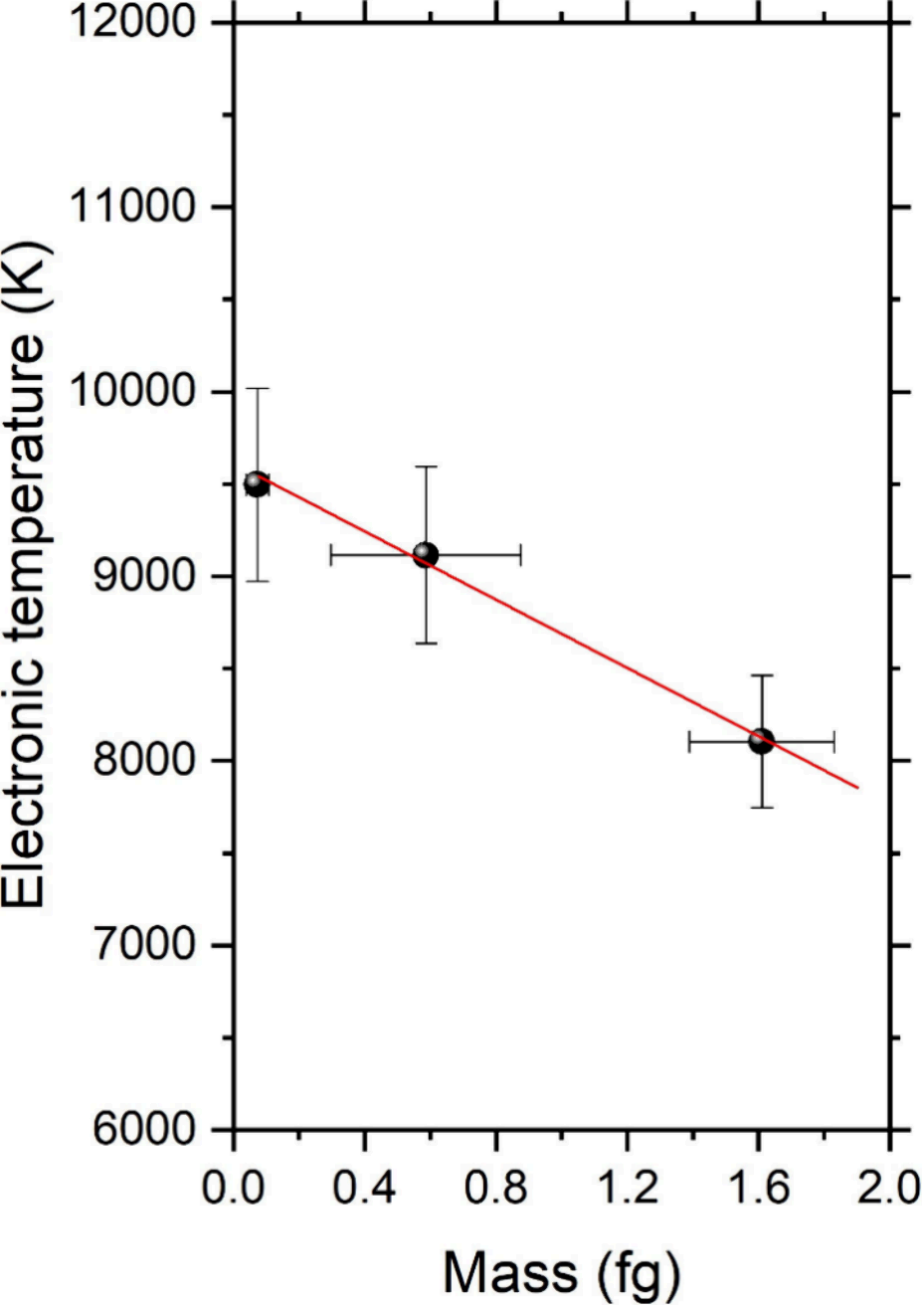
Average electronic temperature calculated for each particle size inspected by using the three Cu(I) lines in the 510–521 nm spectral window. Results are plotted against particle mass, thus confirming the higher energy uptake by larger NPs.

Furthermore, the electronic temperatures (*T*_e_) for each size in ps-LIBS were calculated using the following equation:^33,34^
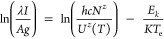
2where λ is the wavelength of the transition, *I* is the integrated net intensity of the emission line, *A* is the transition probability, *g* is the statistical weight of the upper state, *h* is Planck’s constant, *c* is the speed of light, *N^z^* is the total number density of the Cu species in the plasma, *U^z^*(*T*) is the internal partition function for the Cu(I) atoms at temperature *T*, *E_k_* is the energy of the upper state, and *K* is Boltzmann’s constant. Exact values used for the calculation of *T*_e_ are reported elsewhere.^28^ It must be noted that plasma local thermal equilibrium conditions must be assumed for the Boltzmann plot to be applicable. Hence, the used *I* values are those of the three Vis lines at their maximum, i.e., 400 ns (see Figure 5) as Cu atoms are not expected to suddenly increase their *T* beyond this point. Spectral intensities at each particle size were extracted from the averaged LIBS spectra obtained via a data processing protocol. The slope from the Boltzmann plot representing the value of ln(λ*I*/*Ag*) for each of the considered lines yielded the average *T*_e_ results plotted in Figure 7. As observed, *T*_e_ decreased for increasing particle masses: 8104 K (70 nm), 9114 K (50 nm), and 9496 K (25 nm). This experimental result agrees with the fact that larger particles require more energy to be completely atomized. From *T*_e_ and SNR results, it could be concluded that ps*-* excitation is better-suited for quantitative inspection of smaller particles due to its improved atomization capabilities.

## Conclusions

In the present work, ultrafast pulses in the picosecond time regime were used to inspect the composition of single optically trapped nanoparticles both qualitatively and quantitatively. Particles were studied under LIBS spectroscopy and wavelength-resolved plasma imaging, with results being compared to those produced by the more common nanosecond laser excitation. Upon first inspection of spectra produced under both regimes, we found a higher number of analyte-related lines in ps-LIBS. Plasma imaging demonstrated ps*-* plasmas to be more spatially confined with respect to ns ones. This consequence of the reduced thermic effects of ps*-* excitation favored a closer plasma–particle interaction, as opposed to plasma expansion due to energy transferred into air molecules in the medium. Therefore, the ps*-* laser was proved to be more efficient for qualitative NP analysis as the reduced presence of air in the collected spectra complimented the more efficient atom excitation process to result in the apparition of spectral lines missing under ns excitation. On the quantitative side, ps*-* plasmas were demonstrated to be better-suited for the inspection of smaller particle sizes. As larger particles demanded a higher energy input from the laser-induced plasma to dissociate, they caused *T*_e_ depletion proportional to their sizes. This resulted in lower slope and linearity correlation between particle signal and mass. Still, the spectral background reduction owing to decreased thermal effects allows the use of SNR values to enhance the sensitivity of the ultrafast-based approach, thus improving the LOD from that reported using ns. Temporally resolved spectroscopy along wavelength-resolved plasma imaging showed that the mechanisms leading to particle atomization and excitation in ps*-* were similar, yet the particular traits of ps*-* plasmas resulted in earlier onset of particle emission and reduced plasma lifetime. In view of this, spectral signals from NPs inspected using ps*-* excitation should be performed at early stages from plasma onset and using longer integration times to fully exploit the technique’s sensitivity. This work demonstrates the feasibility and validates the use of ultrafast laser pulses as an excitation source for the LIBS inspection of single nanoparticles isolated within atmospheric pressure optical traps at room temperature.

## Supporting Information Available

Figure S1 - Correlation between the signal-to-noise ratios and the nanoparticle mass for each of the inspected Cu NP diameters upon using the spectral signatures at 510.50, 515.29, and 521.74 nm, which were only featured in ps-LIBS featuring their *x*-error bars (PDF)
