# Supplementary material for: Ultrafast Laser Excitation Improves LIBS Performance for the Analysis of Optically Trapped Single Nanoparticles Owing to Characteristic Interaction Mechanisms
Source: Anal Chem. 2023 Sep 20;95(39):14541–50. doi: 10.1021/acs.analchem.3c01376 (PMC10551857; doi:10.1021/acs.analchem.3c01376)

Supporting Information for the manuscript "**Ultrafast laser excitation improves LIBS performance for the analysis of optically trapped single nanoparticles owing to characteristic interaction mechanisms**"

Clara Burgos-Palop[a], Pablo Purohit[a, b], Francisco J. Fortes[a], Javier Laserna[a*]

[a] *UMALASERLAB, Departamento de Química Analítica, Universidad de Málaga, C/Jiménez Fraud 4, Malaga 29010, Spain.*

[b] *Niels Bohr Institute, University of Copenhagen, Blegdamsvej 17, 2100 Copenhagen, Denmark.*

*laserna@uma.es

Contents:

Figure S1: Signal-to-noise ratio vs NP mass for the Cu (I) Vis lines in ps-LIBS…………………………………………………………………………………S2

Figure S1. Nanoparticle mass plotted against their corresponding SNR as in Figure 4B in the main text for the Cu (I) Vis lines present in SP-LIBS spectra generated using ps laser pulses. The present figure also includes the error bars in the x axis. Each featured point is the average net intensity of the corresponding line as extracted from spectra included in Figure 4A. In Figure 4A, the Cu (I) line at 324.75 nm was monitored and observed to consistently show SNR above 3. On the other hand, as seen in the graph, especially in the case of the line at 515.29 nm, the SNR of the concurrent Vis lines fell below the threshold value set to consider a signal as detected (SNR $\geq$3). Furthermore, we observed greater signal variability for these lines, which resulted in x-errors covering mass ranges larger than those specified by the sample manufacturer (which were sound with mass dispersion calculated using the UV line as covered in the main text). Nonetheless, the calculated analytical performance of the method using the 510.50 nm line were on par to that extracted from the use of the 324.75 nm line, thus manifesting the enhanced capabilities of ps-SP-LIBS for nanoinspection.

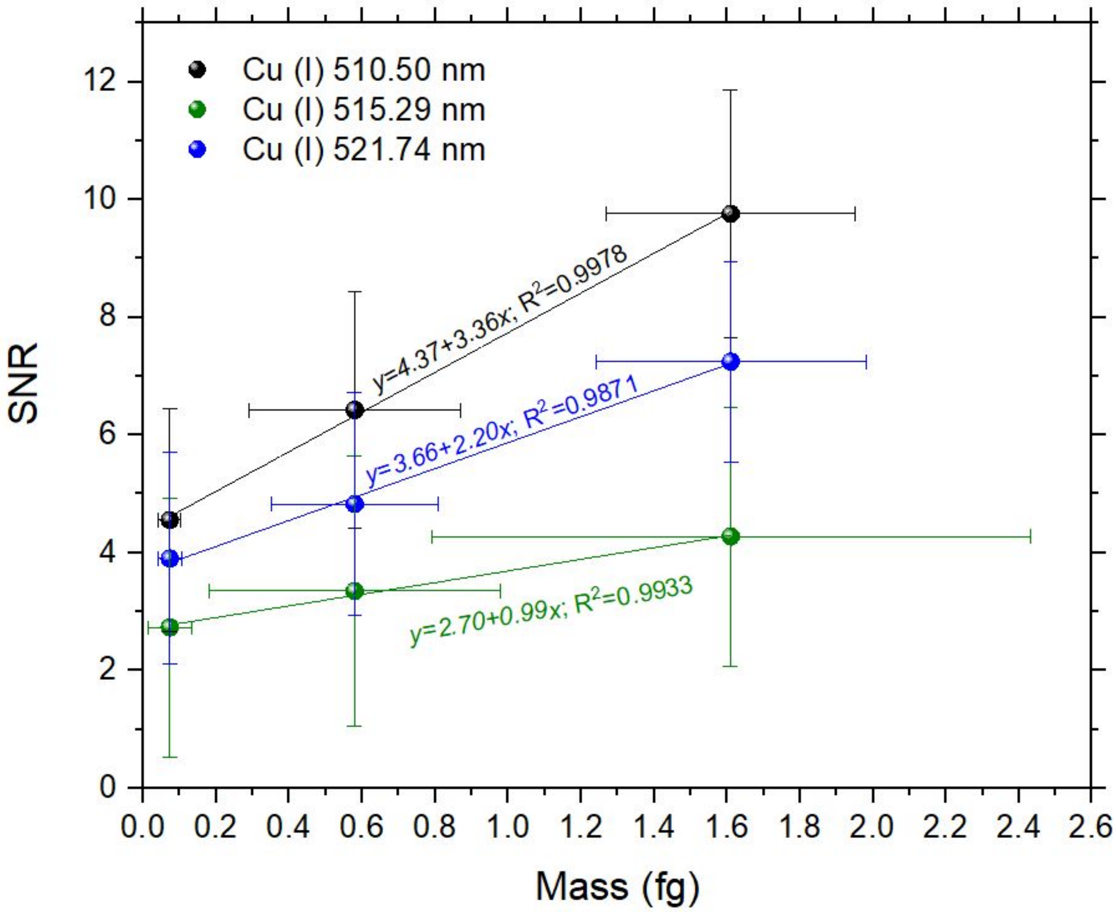

Supplement: Supplementary file 1 — ac3c01376_si_001.pdf [file ac3c01376_si_001.pdf]
